# Identification of Novel Metabolism-Associated Subtypes for Pancreatic Cancer to Establish an Eighteen-Gene Risk Prediction Model

**DOI:** 10.3389/fcell.2021.691161

**Published:** 2021-08-10

**Authors:** Yang Gao, Enchong Zhang, Xiang Fei, Lingming Kong, Peng Liu, Xiaodong Tan

**Affiliations:** ^1^Department of General Surgery, Shengjing Hospital of China Medical University, Shenyang, China; ^2^Department of Urology, Shengjing Hospital of China Medical University, Shenyang, China

**Keywords:** pancreatic cancer, transcriptome, metabolic genes, subtype, risk model

## Abstract

Pancreatic cancer (PanC) is an intractable malignancy with a high mortality. Metabolic processes contribute to cancer progression and therapeutic responses, and histopathological subtypes are insufficient for determining prognosis and treatment strategies. In this study, PanC subtypes based on metabolism-related genes were identified and further utilized to construct a prognostic model. Using a cohort of 171 patients from The Cancer Genome Atlas (TCGA) database, transcriptome data, simple nucleotide variants (SNV), and clinical information were analyzed. We divided patients with PanC into metabolic gene-enriched and metabolic gene-desert subtypes. The metabolic gene-enriched subgroup is a high-risk subtype with worse outcomes and a higher frequency of SNVs, especially in *KRAS*. After further characterizing the subtypes, we constructed a risk score algorithm involving multiple genes (i.e., *NEU2*, *GMPS*, *PRIM2*, *PNPT1*, *LDHA*, *INPP4B*, *DPYD*, *PYGL*, *CA12*, *DHRS9*, *SULT1E1*, *ENPP2*, *PDE1C*, *TPH1*, *CHST12*, *POLR3GL*, *DNMT3A*, and *PGS1*). We verified the reproducibility and reliability of the risk score using three validation cohorts (i.e., independent datasets from TCGA, Gene Expression Omnibus, and Ensemble databases). Finally, drug prediction was completed using a ridge regression model, yielding nine candidate drugs for high-risk patients. These findings support the classification of PanC into two metabolic subtypes and further suggest that the metabolic gene-enriched subgroup is associated with worse outcomes. The newly established risk model for prognosis and therapeutic responses may improve outcomes in patients with PanC.

## Introduction

Pancreatic cancer (PanC) is the fourth most common cause of cancer-related deaths in the United States and accounts for over 227,000 deaths each year globally (Raimondi et al., 2009). The prevalence and incidence have increased in the past few years and this trend is expected to continue (Oshi et al., 2020). The etiology of PanC is complex and multiple risk factors have been reported, including genetic factors, lifestyle factors, prior diseases, infections, and occupational exposure (Vincent et al., 2011). Universal treatment strategies fail to manage PanC owing to its heterogeneity at the level of molecular phenotypes and pathological and clinical properties (Makohon-Moore and Iacobuzio-Donahue, 2016). The complicated molecular characteristics render histopathology insufficient for clinical decisions and prognostic analyses (Collisson et al., 2019). The Cancer Genome Atlas (TCGA) Research Network completed integrated genomic, transcriptomic, and proteomic profiling of 150 pancreatic ductal adenocarcinoma specimens. They revealed a complex molecular landscape of pancreatic ductal adenocarcinoma and provided a roadmap for precision medicine (Cancer Genome Atlas Research Network, 2017). Two large researches using PanC samples recently reported gene expression subtypes of PanC, extending the subtypes previously described by Collisson et al. (2011), Bailey et al. (2016), Martinez-Useros et al. (2021). Sinkala et al. employed transcriptomic, copy number alteration and mutation profiling datasets from PanC patients together with data on clinical outcomes to show that the three PanC subtypes each display distinctive aberrations in cell signaling and metabolic pathways (Sinkala et al., 2018). Sinkala et al. also identified another two PanC subtypes and biomarker sets that can be used to accurately and sensitively classify novel pancreatic tumors (Sinkala et al., 2020).

Cancer metabolism plays an important role in oncogenesis and progression, and metabolic activity could be considered a cancer hallmark. There is compelling evidence that tumors reprogram cellular pathways for nutrient retrieval and consumption, allowing cancer cells to meet the demands of bioenergy generation, biosynthesis, and redox reactions (DeBerardinis and Chandel, 2016; Reina-Campos et al., 2017). Orchestrating adaptations of malignant cells in the hypoxic environment, cancer metabolism also presents heterogeneity and plasticity, even within the same malignancy, with differences among subpopulations, such as cell clusters with distinct rates of division (Birsoy et al., 2014; LeBleu et al., 2014; Yoshida, 2015). The dominant genetic alterations in pancreatic ductal adenocarcinoma are *KRAS* oncogene mutations (Buscail et al., 2020). In PanC cells, *KRAS* promotes extracellular glucose uptake by upregulating the glucose transporter GLUT1 and hexokinase (Ying et al., 2012). Furthermore, the hypoxic environment in PanC induces the stabilization of the transcription factor hypoxia-inducible factor 1, which promotes cancer cell adaptation to hypoxic conditions and is associated with an unfavorable prognosis (Jin et al., 2020). Metabolic mechanisms, involving micropinocytosis, in PanC can predict therapeutic efficacies (Commisso et al., 2013). Accordingly, studies of metabolic diversity and the identification of reliable prognostic markers for PanC are urgently needed.

Machine learning approaches can provide insights into disease diagnosis and treatment based on large amounts of data (Rajkomar et al., 2019). In PanC, Zhu et al. used a bioinformatics approach to identify *TMPRSS*4, *SERPINB5*, *SLC6A14*, *SCEL*, and *TNS4* as diagnostic biomarkers in PanC (Cheng et al., 2019). Machine learning provides depth and width in PanC investigations and may facilitate precision medicine (Herbst and Zheng, 2019). Another interesting study identified pancreatic ductal adenocarcinoma morphological subtypes using machine learning, providing new ideas for PanC management (Kalimuthu et al., 2020). In our study, we hypothesized that PanC could be divided into subtypes with distinct metabolic involvement, and genes related to these subtypes could be utilized to predict outcomes. In particular, we identified and characterized two subgroups with metabolic-enriched and metabolic-desert characteristics based on genes from Molecular Signatures Database v7.2. Finally, we trained and validated a risk model based on these metabolic classifications.

## Materials and Methods

### Data Acquisition and Processing

**FIGURE 1:**
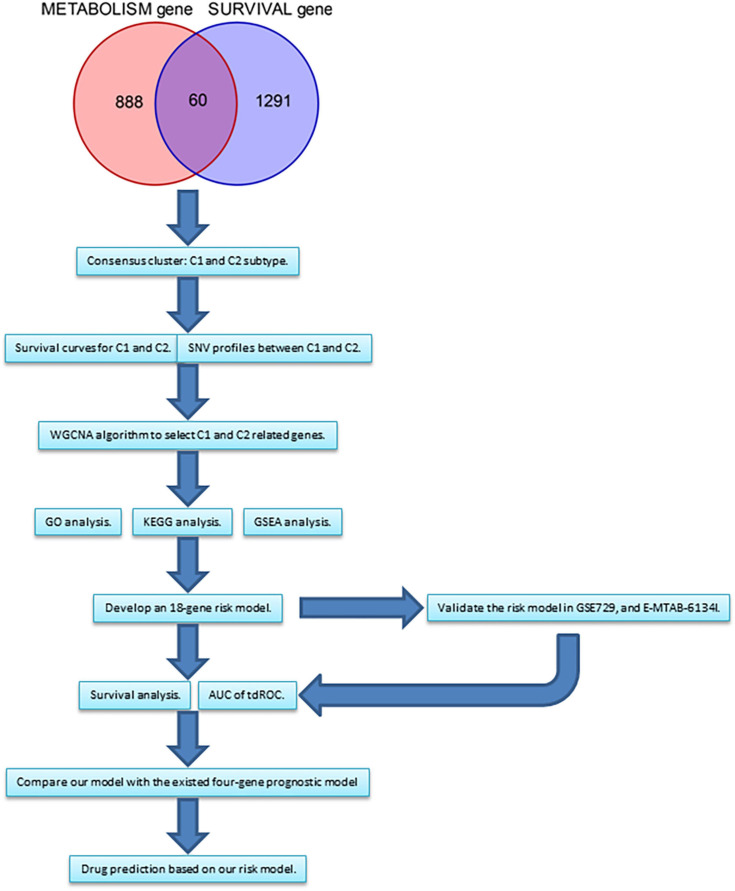
The flow chart summarizing the process of this study. WGCNA, weighted correlation network analysis. GO, gene ontology. KEGG, Kyoto Encyclopedia of Genes and Genomes.

Transcriptome profiles, simple nucleotide variant (SNV), and clinical data for 171 patients with PanC were downloaded from The Cancer Genome Atlas (TCGA)^1^ (Blum et al., 2018). All analyses of TCGA data were performed using R 3.6.3. Pathological and clinical information is shown in Table 1. A risk prediction model was developed. Two additional datasets were obtained from ArrayExpress^2^ and Gene Expression Omnibus^3^ to validate the effectiveness of the model. The details of these two datasets are provided in Table 2. One flow chart was displayed in Figure 1 to summarize the process of this study.

**TABLE 1:** The disease-related clinical information of patients with pancreatic cancer included in the study.

Characteristics	Value
Patients(n)	171
Age(year), median(IQR)	65.7(57.2–73.3)
**Gender, n(%)**	
female	78(45.6%)
male	93(54.4%)
**Pathological M, n(%)**	
M0	77(45.0%)
M1	4(2.3%)
NA	90(52.7%)
**Pathological T, n(%)**	
T1 + T2	28(16.4%)
T3 + T4	141(82.5%)
NA	2(1.1%)
**Pathological N, n(%)**	
N0	47(27.5%)
N1 + N1b	119(69.6%)
NA	5(2.9%)
**Pathological stage, n(%)**	
Stage I	19(11.1%)
Stage II	142(83.0%)
Stage III + IV	7(4.1%)
NA	3(1.8%)
**Outcome, n(%**)	
Dead	91(46.8%)
Alive	80(53.2%)

*IQR, interquantile range; NA, not analyzed.*

**TABLE 2:** Information of the two publicly available independent validation datasets.

Dataset	Sample size	Transcriptome platform	Tissue
GSE729	125	Agilent-014850 Whole Human Genome Microarray	Fresh frozen
E-MTAB-6134	288	Affymetrix Human Genome U219 Array	Fresh frozen and formalin-fixed paraffin-embedded

### Identification of Metabolism-Based Subtypes

To filter out genes not associated with prognosis in PanC, a global survival analysis for each gene was performed via log-rank test and Cox regression using the survival R package. Thereafter, metabolic genes were extracted from Molecular Signatures Database v7.2^4^ (Liberzon et al., 2015). The overlap between survival-associated genes and metabolism-related genes was obtained. Finally, the expression levels of intersecting genes were used for a consensus clustering analysis to identify novel subtypes using the ConsensusClusterPlus R package. Default parameters were used (Wilkerson and Hayes, 2010). As determined using the ConsensusClusterPlus R package, the curve of the cumulative distribution function (CDF) and area under the CDF were used to choose the best k-value for the number of clusters. To evaluate differences between novel subtypes, a principal component analysis (PCA) was used. Next, survival curves were generated to evaluate the prognostic value of subtypes using the survminer and survival R packages. Expression levels of ten randomly selected genes used as inputs in the clustering analysis were examined to further characterize the different subtypes. The relationship (possible overlaps in patient samples) between the transcription subtypes that have been previously defined and the subtypes identified by us was shown via circle plot (Collisson et al., 2011; Moffitt et al., 2015; Bailey et al., 2016). Finally, tumor purity information acquired from TCGA Research Network was compared between two subtypes by *t*-test (Cancer Genome Atlas Research Network, 2017).

### SNVs in the Subtypes

Using SNV data obtained from TCGA, the mutation frequencies of all genes were calculated. Thereafter, the ten genes with the highest mutation rates were utilized to evaluate the difference between subtypes using the GenVisR R package. Differences were visualized using waterfall plots. Next, genes with significant differences in SNV statuses were identified and the relationships between SNV statuses and transcript levels were determined. Finally, survival curves based on SNV statuses of these genes were generated using the survival and survminer R packages.

### Weighted Correlation Network Analysis of Genes Related to Subtypes

Although novel subtypes were identified using the ConsensusClusterPlus package based on the expression levels of selected genes, this analysis did not reveal the precise genes related to each subtype and the sample size of genes was not sufficient for a network analysis. Therefore, a weighted correlation network analysis (WGCNA) was employed to identify gene co-expression modules related to the characteristics of the subtypes (Langfelder and Horvath, 2008) using the R package WGCNA. As recommended in the package instructions, the soft threshold was set to 7. For gene module fusion, the cutoff value was set to 0.25, as described previously (Kong et al., 2020).

### Identification of Hub Genes Based on Subtypes

Based on the WGCNA, genes that were highly related to the high-risk subtype were obtained. These genes were used as inputs for an enrichment analysis using Metascape^5^ (Kong et al., 2020) using default parameters. According to the annotations in Metascape, for each given gene list, protein-protein interaction enrichment analysis has been carried out, and if the network contains between 3 and 500 proteins, the Molecular Complex Detection (MCODE) algorithm would be applied to identify densely connected network components. Pathway and process enrichment analysis has been applied to each MCODE component independently, and the five best-scoring terms by p-value have been selected.

### Enrichment Analyses of Subtypes

To further clarify the crucial processes activated in the high-risk subtype, a GSEA was performed. Hallmark gene sets were used as the background set (Liberzon et al., 2011). Fold change values were obtained for the comparison between high-risk and low-risk subtypes using the DESeq2 R package (Love et al., 2014). Next, the clusterProfiler R package was used to perform the GSEA (Yu et al., 2012). Furthermore, Gene ontology (GO) and Kyoto Encyclopedia of Genes and Genomes (KEGG) enrichment analyses directly based on the genes found up-regulated in C1 and also on the genes up-regulated in C2 were conducted via clusterProfiler R package (Yu et al., 2012).

### Development of a Risk Prediction Model Based on Metabolism-Related Subtypes

A risk prediction model was developed based on the metabolism-associated subtypes. A group of genes associated with the high-risk subgroup was used to develop a risk model by the least absolute shrinkage and selection operator (Lasso), a frequently adopted method (Zhang E. et al., 2020; Zhang H. et al., 2020; Zhang M. et al., 2020). The set of 171 patients from TCGA was divided into a training group (*n* = 120) and internal validation group (*n* = 51) using the caret R package. This package ensures that patients are equally divided into two distinct groups. Subsequently, data for patients in the training group were used to construct a risk model by Lasso. The regression analysis was performed using the glmnet R package with default parameter settings. The model was validated using the training group, internal validation group, and two additional external validation data sets. A survival analysis and time-dependent receiver operating characteristic (tdROC) curves were used as indicators for model effectiveness. Finally, the risk score of our model was compared to that of an existed four-gene prognostic model in term of tdROC curves (Yan et al., 2020).

### Univariate and Multivariate Cox Regression Analyses

To determine the independent prognostic value of the model, univariate and multivariate Cox regression analyses of the model and other clinical or pathological variables were performed. A univariate Cox regression was performed first. Statistically significant variables in the univariate analysis were included in the multivariate analysis, as described previously (Kong et al., 2020). These analyses were performed using the survival R package.

### Drug Target Prediction

Using the CTRP2.0 and PRISM databases, the sensitivities of various agents for patients with high risk scores based on gene expression levels were predicted via ridge regression. The pRRophetic R package was used for prediction (Geeleher et al., 2014). Components with significantly lower areas under the dose–response curve (dr-AUC) in high-risk patients than in low-risk patients were selected. Thereafter, Spearman’s correlation coefficients for the relationship between dr-AUC and risk score were determined. Components with significantly negative rho values (i.e., less than −0.3) were obtained.

### Statistical Analysis

All statistical analyses were performed using R version 3.6.3. In a global survival of all genes, log-rank test and Cox regression were used, setting *P* < 0.01 as the cut-off value for significance. In all other analyses, *P* < 0.05 was the threshold for statistical significance.

## Results

### Identification of Two Metabolism-Associated Subtypes

**FIGURE 2:**
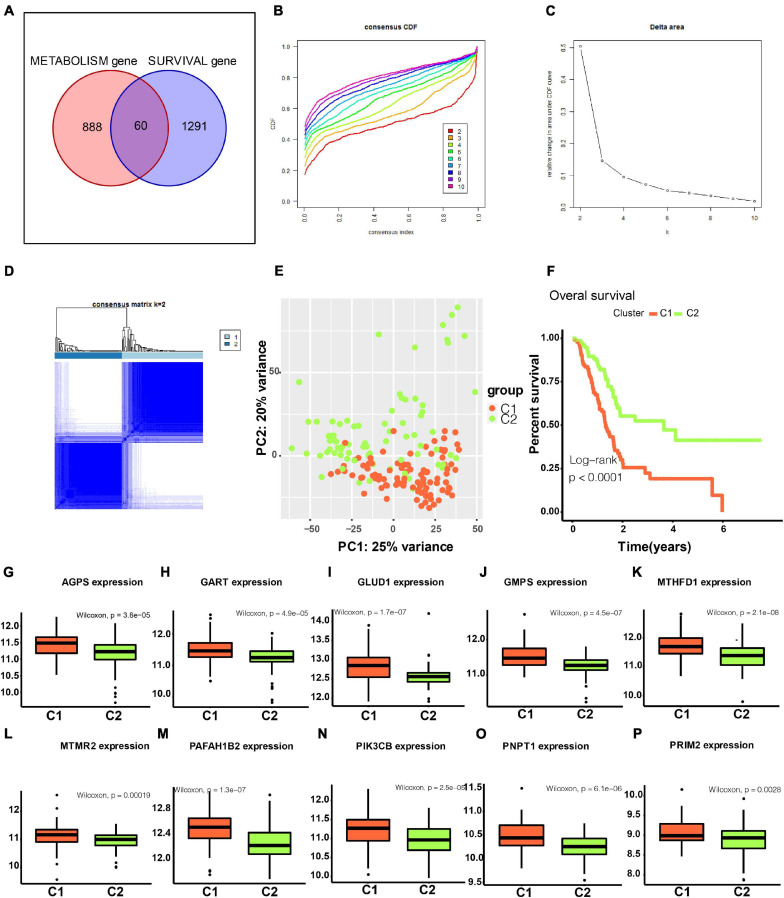
Identification of two metabolism-associated subtypes. **(A)** In total, 60 genes were identified in the intersection between survival-associated genes and metabolism-related genes. **(B)** CDF curve for different k-values (representing the number of clusters in the consensus clustering analysis). When the optimal k-value is reached, the area under the CDF curve does not increase significantly as the k-value increases. **(C)** Relative change in the area under the CDF curve for different values of k. **(D)** Consensus matrix obtained when *k* = 2. Consistency values range from 0 to 1, where 0 indicates that genes never cluster together (white) and 1 indicates that they always cluster together (dark blue). **(E)** PCA showed a significant difference between subtypes. **(F)** Survival curves for patients with different subtypes. **(G–P)** Boxplot depicting expression levels of some metabolic genes in the C1 and C2 subtypes. CDF, cumulative distribution function. PCA, principal components analysis. *P* < 0.05 is defined as statistically significant.

In total, 60 genes were included in a consensus clustering analysis (Figure 2A). As shown in Figure 2B, *k* = 2 was the optimal value. As shown in Figure 2C, the area under the CDF did not increase significantly for *k* > 2, further indicating that the optimal k-value was 2. Figure 2D shows the consensus matrix for two subtypes (referred to as C1 and C2). A PCA further supported the difference between the two subtypes (Figure 2E). A survival analysis indicated that patients with the C1 subtype had a worse prognosis than that of patients with the C2 subtype (Figure 2F). As shown in Figures 2G–P, the C1 subtype was highly enriched for metabolism-related genes. We found that the classical subtype identified by Collisson et al. was mainly a subset of C1 subtype (Supplementary Figure 1A) (Collisson et al., 2011). Furthermore, progenitor and squamous subtypes via Bailey et al. were mainly subsets of C1 subtype (Supplementary Figure 1A) (Bailey et al., 2016). Finally, high tumor purity might be the driver factor for the characteristics of C1 subtype (Supplementary Figure 1B, *t*-test *P* < 0.001).

### Difference in SNVs Between Subtypes

**FIGURE 3:**
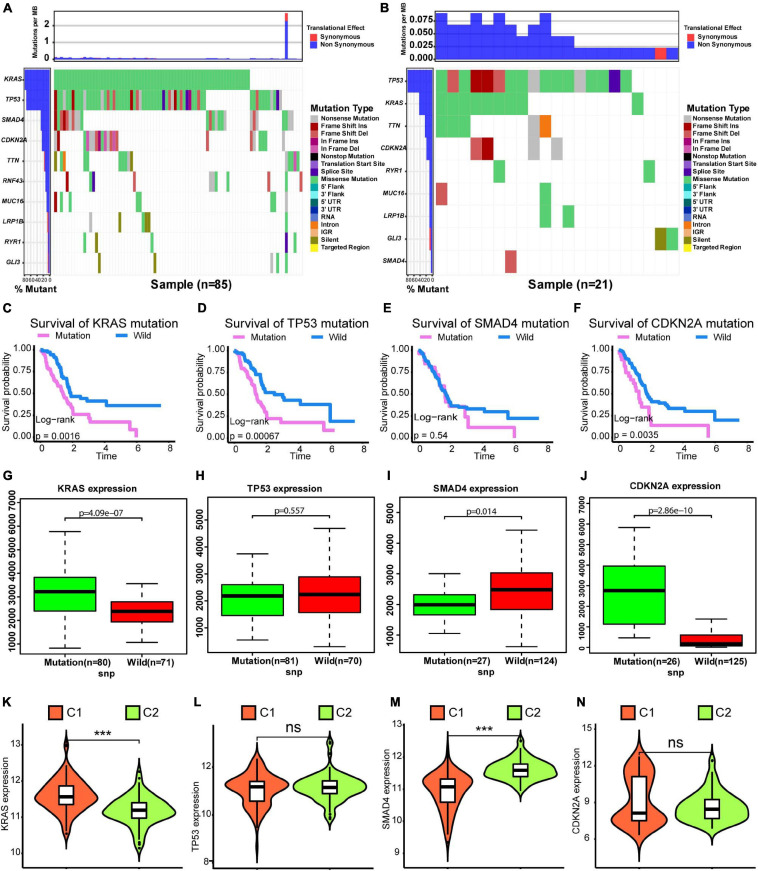
Differences in SNVs between C1 and C2 subtypes. **(A)** Waterfall plot for the C1 subtype. **(B)** Waterfall plot for the C2 subtype. **(C–F)** Kaplan–Meier survival analysis of patients with mutations in *KRAS*, *TP53*, *SMAD4*, and *CDKN2A*. *P-*values obtained via log-rank tests are shown. **(G–J)** Boxplots illustrating correlations between gene expression levels and SNVs for *KRAS*, *TP53*, *SMAD4*, and *CDKN2A*. **(K–N)** Violin plots exhibit differences in expression levels of *KRAS*, *TP53*, *SMAD4*, and *CDKN2A* between C1 and C2 subtypes. Wilcoxon test *P*-values are shown. SNV: simple nucleotide variation. *P* < 0.05 was defined as statistically significant.

We compared SNVs in the two subgroups. As shown in Figures 3A,B, the proportion of SNVs was higher in C1 than in C2. *KRAS*, *TP53*, *SMAD3*, and *CDKN2A* had high SNV frequencies. There were 69 patients harbored *KRAS* mutation in C1 subtype, and 10 patients harbored *KRAS* mutation in C2 subtype. There were 65 patients harbored *TP53* mutation in C1 subtype, and 17 patients harbored *TP53* mutation in C2 subtype. We found that there were lots of patients harbored *TTN* mutations in C1 and C2 subtypes. However, *TTN* was not significantly mutated based on the MutSigCV algorithm on Firebrowse database^6^ (Broad Institute TCGA Genome Data Analysis Center, 2016). We found that patients with SNVs of *KRAS*, *SMAD3*, and *CDKN2A* exhibited a worse overall survival (Figures 3C–F). Finally, we found that patients with SNVs in *KRAS* and *CDKN2A* exhibited higher gene expression levels than those in patients without SNVs. However, the SNV in *SMAD4* was correlated with a lower level of gene expression. SNVs in *TP53* did not show a significant relationship with the level of gene expression (Figures 3G–J). Furthermore, *KRAS* expression was significantly higher in the C1 subtype than in the C2 subtype (Figure 3K, Wilcoxon test *P* < 0.001). *SMAD4* expression was significantly lower in the C1 subtype than in the C2 subtype (Figure 3M, Wilcoxon test *P* < 0.001). However, there was no significant difference for levels of TP53 and CDKN2A between C1 and C2 subtypes (Figures 3L,N). Generally, alterations in gene expression levels in the C1 subtype could be explained by the SNV status.

### Identification of Gene Networks Related to the C1 and C2 Subtypes by WGCNA

**FIGURE 4:**
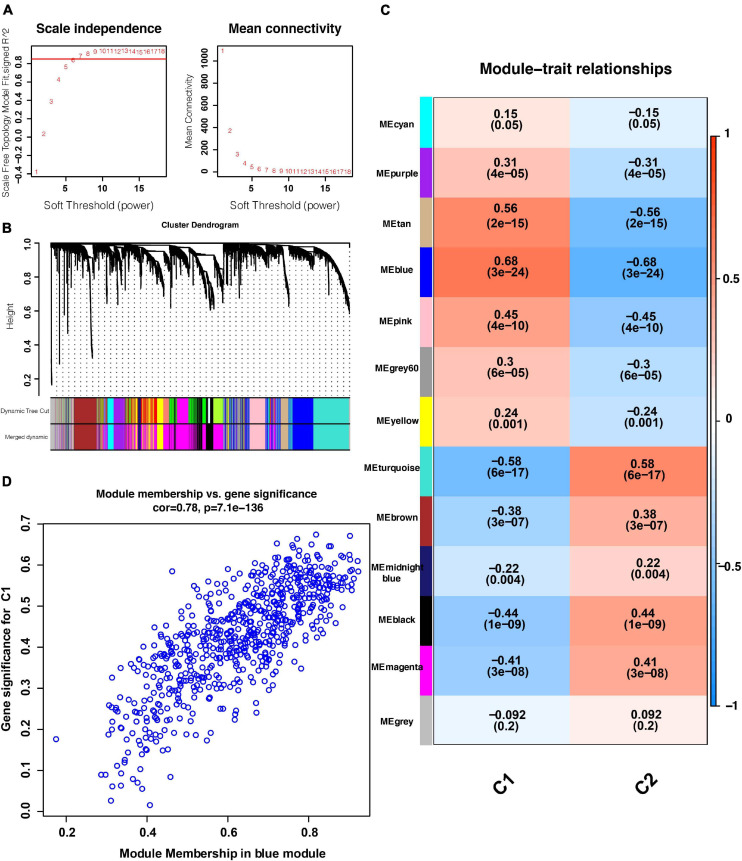
WGCNA results. **(A)** Relationship between the soft threshold and TOM-based dissimilarity (left). Relationship between the soft threshold and mean connectivity (right). **(B)** After cutting and merging, 13 gene modules were finally generated. **(C)** Heat map of the correlations between gene modules and phenotypes. **(D)** Scatter plot depicting the correlations between gene significance and module membership of genes in the blue network. WGCNA: weighted correlation network analysis. TOM, topological overlap matrix. *P* < 0.05 is defined as statistically significant.

As shown in Figure 4A, when the soft threshold was set to 7, the gene networks satisfied both a high degree of internal connectivity and high gene similarity. The top 5000 genes with a high degree of variation were divided into gene networks. Gene networks whose genes showed no significant differences were merged and finally 13 networks were identified (Figure 4B). Next, correlations between networks and phenotypes were evaluated. The blue network shown in Figure 4C was most closely related to the C1 subtype and the turquoise network was most related to the C2 subtype. The significance (i.e., the extent to which it represents the corresponding phenotype) and module membership of genes in the blue network are shown in Figure 4D, and these parameter values exhibited a strong positive correlation.

### Enrichment Analysis and Hub Gene Selection

**FIGURE 5:**
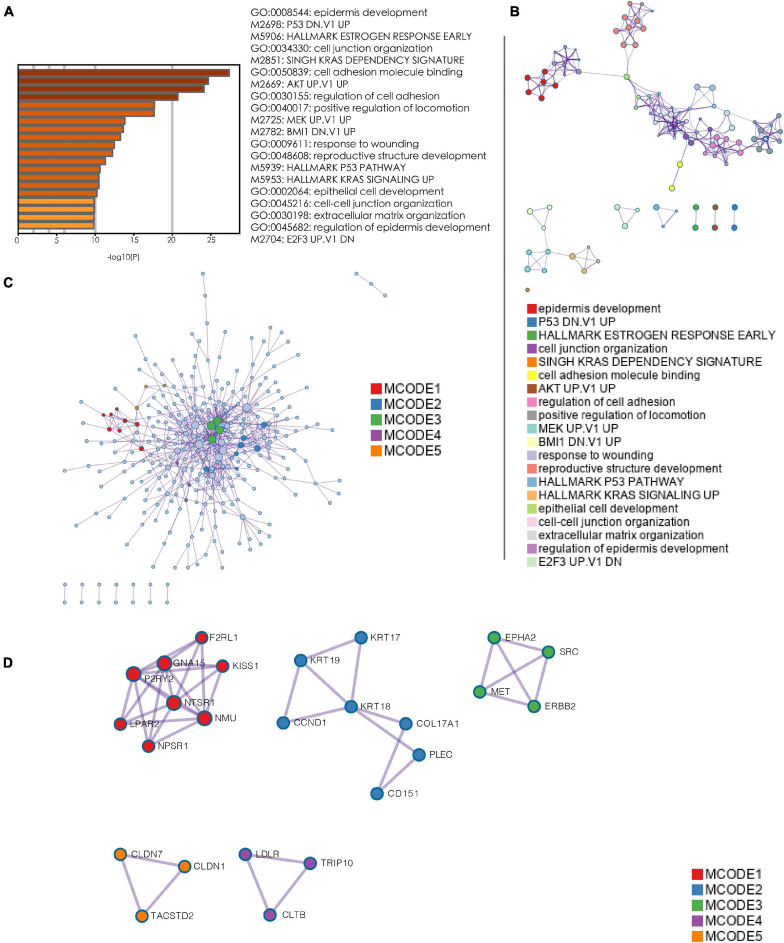
Enrichment analysis and hub gene selection. **(A)** Bar plot depicting the results of an enrichment analysis of genes in the blue module. **(B)** Interaction network with enrichment terms. **(C)** Protein–protein interaction networks for genes generated using MCODE. **(D)** Five key protein–protein interaction networks identified using MCODE. (MCODE: molecular complex detection).

Owing to the worse prognosis of the C1 subtype, we focused on the network that was most strongly correlated with this subtype (i.e., the blue network in Figure 4). We extracted genes in this network and used them as inputs for an analysis using the Metascape database. As determined via an enrichment analysis, the downregulation of P53 signaling and activation of KRAS, AKT, and MEK signaling were enriched in the blue network, suggesting that these biological activities were likely associated with the C1 subtype (Figures 5A,B). Thereafter, hub genes were selected using the MCODE algorithm. As shown in Figure 5C, five sub-networks were selected. *KRT18* was identified as a hub gene with the potential to affect the development of PanC (Figure 5D).

### GSEA of the High-Risk Subtype

**FIGURE 6:**
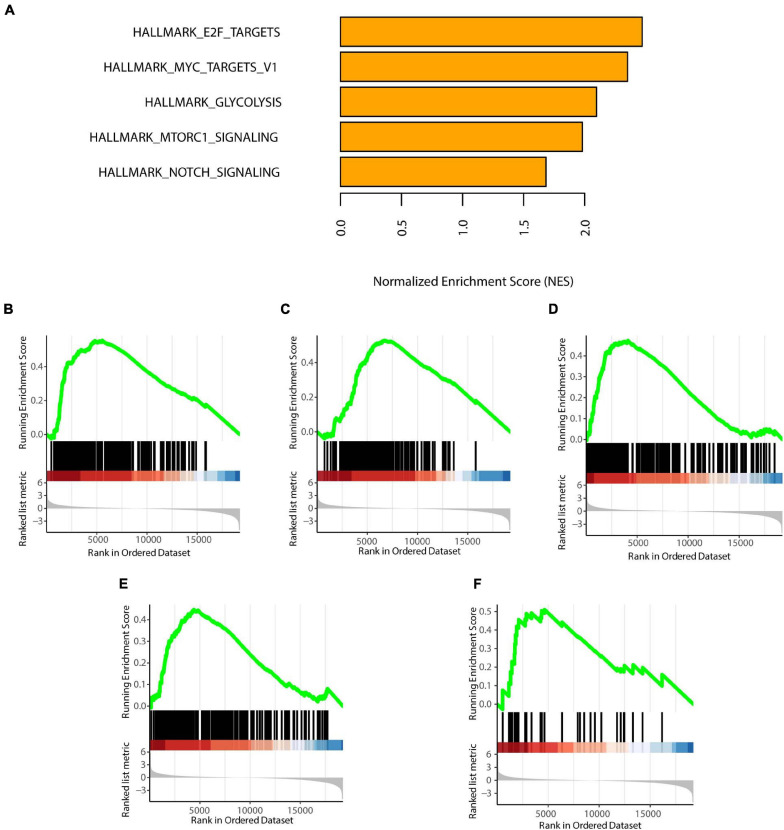
GSEA of the two subtypes. **(A)** Top five enrichment terms (ranked in descending order of NES). **(B)** HALLMARK_E2F_TARGETS. **(C)** HALLMARK_MYC_TARGETS_V1. **(D)** HALLMARK_GLYCOLYSIS. **(E)** HALLMARK_MTORC1_SIGNALING. **(F)** HALLMARK_NOTCH_SIGNALING. NES: normalized enrichment scores. *P* < 0.05 was defined as statistically significant.

We selected statistically significant gene sets in the GSEA and ranked these sets according to normalized enrichment scores (NES). The top five results are displayed in Figures 6A–F. We found that HALLMARK_E2F_TARGETS, HALLMARK_MYC_TARGETS_V1, HALLMARK_GLYCOLYSI S, HALLMARK_MTORC1_SIGNALING, and HALLMARK_ NOTCH_SIGNALING were all activated in the C1 subtype. These biological activities were all cancer-promoting processes, consistent with the high-risk feature of the C1 subtype (Sancho et al., 2015; Di Malta et al., 2017; Qin et al., 2019; Katoh and Katoh, 2020; Oshi et al., 2020). Furthermore, the GO and KEGG enrichment results were showed in Supplementary Figure 2.

### Construction and Validation of an 18-Gene Risk Model

**FIGURE 7:**
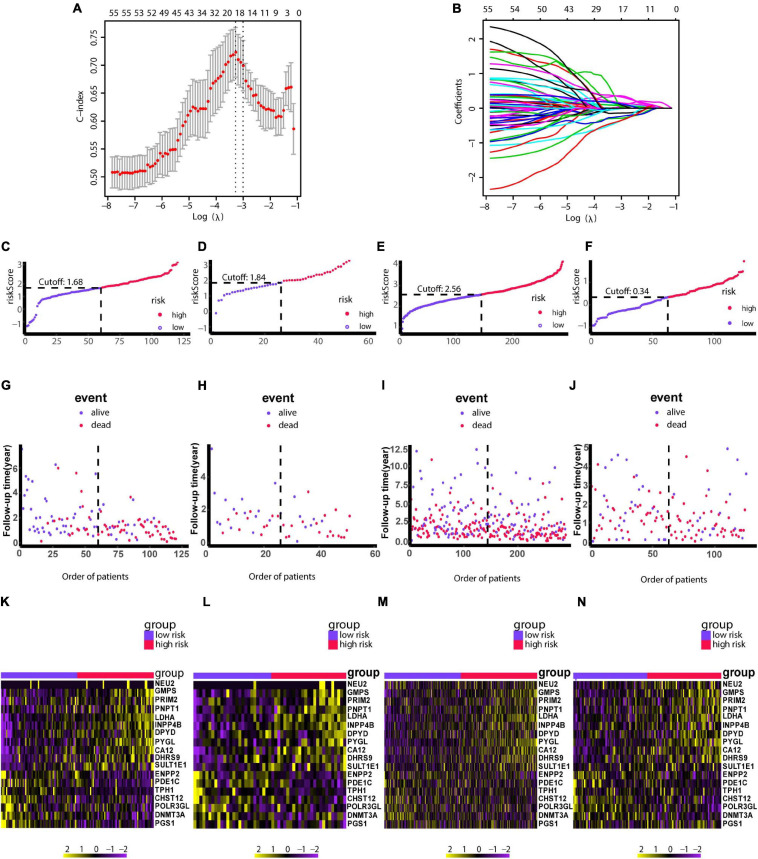
Construction of the risk model using LASSO. **(A)** Cross-validation based on the C-index to determine the optimal number of genes in the model. **(B)** Genes in different combinations and corresponding coefficients. **(C,G,K)** Patients in the training set (*n* = 120) were arranged in ascending order of risk scores. **(D,H,L)** Patients in the internal validation set (*n* = 51) were arranged in ascending order of risk scores. **(E,I,M)** Patients in the GSE729 data set (*n* = 125) were arranged in ascending order of risk scores. **(F,J,N)** Patients in the E-MTAB-6134 data set (*n* = 288) were arranged in ascending order of risk scores. **(C–F)** Patients were divided into different risk levels according to median risk scores in their respective data sets. **(G–J)** Relationships between survival outcomes and risk levels. Low-risk patients are shown on the left side of the dotted line and high-risk patients are shown on the right side. **(K–N)** Heat maps for genes in the model. LASSO, least absolute shrinkage and selection operator. *P* < 0.05 was defined as statistically significant.

**FIGURE 8:**
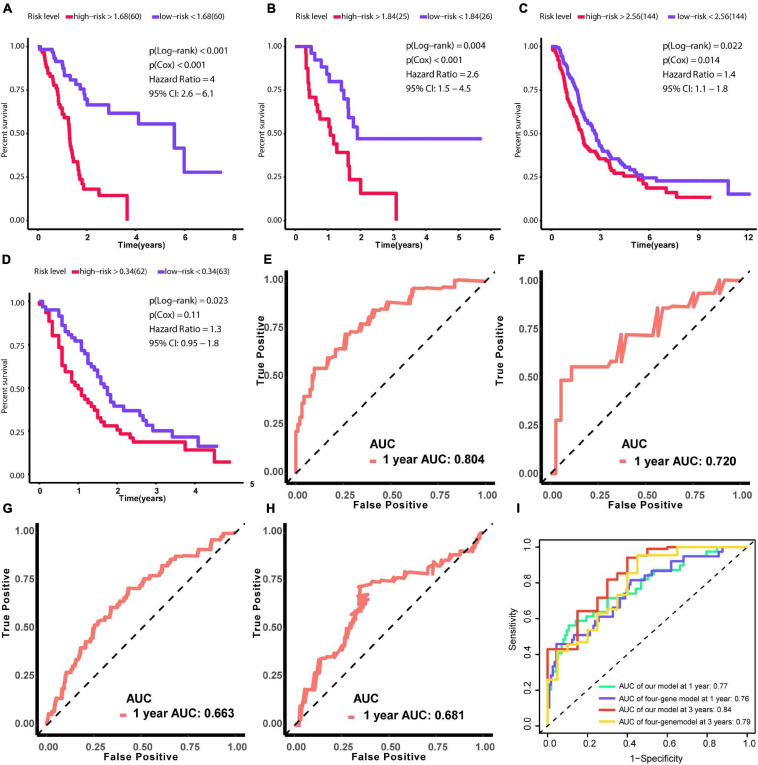
Verification of the effectiveness of the prognostic signature. **(A–D)** Survival analysis based on Kaplan–Meier curves. **(E–H)** ROC curve for 1-year follow-up. **(A,E)** Results obtained using the training set. **(B,F)** Results obtained using the internal validation set. **(C,G)** Results obtained using GSE729. **(D,H)** Results obtained using E-MTAB-6134. **(I)** The comparison between our model and the existed four-gene model in term of tdROC curves. (AUC, area under curve; tdROC, time-dependent receiver operating characteristic). The clinical outcome endpoint was overall survival. *P* < 0.05 was defined as statistically significant.

Due to patients in C1 subtype had the worst prognosis, we used the genes in blue network, which is most related to C1 subtype, to train the risk model via Lasso regression using the training group. During the selection of genes for the model, the C-index obtained by cross-validation was used for feature selection. Figure 7A showed the corresponding C-index values for models with different gene combinations. The X-axis is the number of genes in the corresponding model. As shown in Figure 7A, a combination of 18 genes showed the highest C-index. Figure 7B shows the coefficients for each gene and combination. Finally, we developed an 18-gene risk model, providing a basis for determining risk levels of patients as follows: Risk score = NEU2 × 0.390 + GMPS × 0.350 + PRIM2 × 0.144 + PNP T1 × 0.154 + LDHA × 0.0004 + INPP4B × 0.011 + DPYD × 0.187 + PYGL × 0.363 + CA12 × 0.098 + DHRS9 × 0.080 + SULT1E1 × 0.158 - ENPP2 × 0.039 - PDE1C × 0.297 - TPH1 × 0.042 - CHST12 × 0.145 - POLR3GL × 0.421 - DNMT3A × 0.456 - PGS1 × 0.267. Next, patients in the training group (*n* = 120), internal validation group (*n* = 51), GSE729 data set (*n* = 125), and E-MTAB-6134 data set (*n* = 288) were ranked according to risk scores (Figures 7C–N). The expression levels of these 18 genes were globally evaluated in the four groups and the stability of the results were robust. Thereafter, survival and tdROC analyses were used to evaluate the effectiveness of the risk model in four data sets. As shown in Figures 8A–D, patients with higher risk scores had a worse overall survival. In the tdROC, the AUC values all exceeded 0.65, indicating good model performance (Figures 8E–H). Finally as shown in Figure 8I, we found that our model performed better than the existed four-gene prognostic model in TCGA-PAAD cohort (*n* = 171) (AUC of our model at 1 year: 0.77; AUC of four-gene model at 1 year: 0.76; AUC of our model at 3 years: 0.84; AUC of four-gene model at 3 years: 0.79).

### Prognostic Value of the Model

**FIGURE 9:**
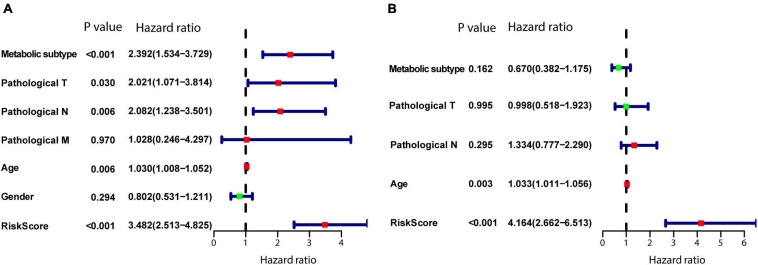
Establishment of the model as an independent prognostic factor. **(A)** Univariate Cox regression. **(B)** Multivariate Cox regression. *P* < 0.05 was defined as statistically significant.

To determine whether the model is an independent predictor of prognosis, we performed univariate and multivariate Cox regression analyses. In the univariate Cox regression analysis, metabolism-associated subtype, pathological T, pathological N, age, and risk score from the model were risk factors for prognosis (Figure 9A). Next, these variables were included in a multivariate Cox regression analysis, which showed that age and risk score are independent predictors of prognosis (Figure 9B).

### Target Drug Prediction for High-Risk Patients

**FIGURE 10:**
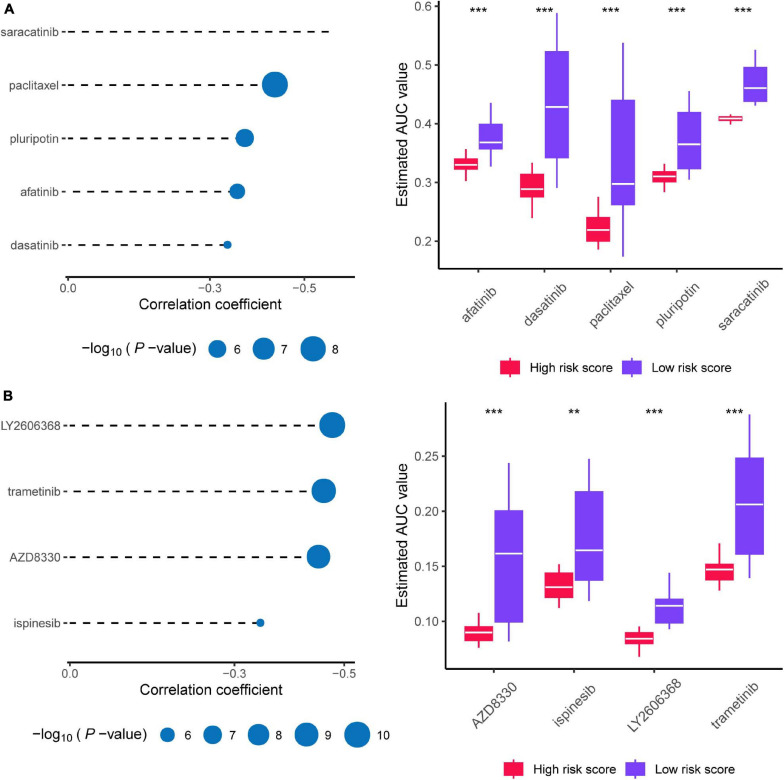
Prediction of candidate agents with higher drug sensitivity in patients with high risk score. **(A)** The results of Spearman’s correlation analysis and differential drug response analysis of three CTRP-derived compounds. **(B)** The results of Spearman’s correlation analysis and differential drug response analysis of three PRISM-derived compounds. Note that lower values on the y-axis of boxplots imply greater drug sensitivity. * means *P* < 0.05, ** means *P* < 0.01, *** means *P* < 0.001, ns means *P* > 0.05, and *P* < 0.05 is defined as statistically significant.

As shown in Figure 10A, afatinib, dasatinib, paclitaxel, pluripotin, and saracatinib were predicted with high sensitivity in patients with high risk scores (Spearman correlation test rho < -0.3, Spearman correlation test *P* < 0.001, and Wilcoxon test *P* < 0.001). As shown in Figure 10B, AZD8330, ispinesib, LY2606368, and trametinib showed high sensitivity for patients with high risk scores (Spearman correlation test rho < –0.3, Spearman correlation test *P* < 0.001, and Wilcoxon test *P* < 0.01). Collectively, we predicted nine target drugs for high-risk patients; paclitaxel, AZD8330, and LY2606368 showed the highest effectiveness.

## Discussion

Recent studies have offered ample insight into multi-omics landscapes to define PanC subtypes (Noll et al., 2016; Mishra and Guda, 2017; Puleo et al., 2018; Lomberk et al., 2018). Lomberk et al. performed multi-parametric integrative analyses of chromatin immunoprecipitation-sequencing on multiple histone modifications, RNA-seq, and DNA methylation to define epigenomic landscapes for PanC subtypes, which can predict their relative aggressiveness and survival. Puleo et al. identified five PanC subtypes, based on features of cancer cells and the tumor microenvironment. Kumar Mishra et al. performed genome-scale methylome analysis of PanC data from TCGA and provided a strong basis for future work on the molecular subtyping of epigenetic regulation in PanC. Using data obtained from multiple platforms (TCGA, GSE729, and E-MTAB-6134), we identified novel PanC subtypes and prognostic signatures based on cancer metabolism. Patients with PanC from the TCGA dataset were divided into metabolic-enriched and metabolic-desert groups, C1 and C2, respectively. Multiple metabolic genes (*AGPS*, *GART*, *GLUD1*, *GMPS*, *MTHFD1*, *MTMR2*, *PAFAH1B2*, *PIK3CB*, *PNPT1*, and *PRIM2*) related to aggressiveness and poor outcomes in cancer showed higher expression levels in the C1 group than in the C2 group. Alkylglyceronephosphate synthase (AGPS), an essential metabolic enzyme in cancer, is involved in the synthesis of ether lipids and promotes the aggressive features of multiple malignances (Benjamin et al., 2013). Widely recognized as a prognostic factor in many cancers, glucose transporter-1 (GLUT1) is associated with both a poor prognosis and drug resistance in cancers, such as PanC, cervical cancer, and breast cancer (Nagarajan et al., 2017; He et al., 2019; Kim and Chang, 2019). Other metabolic genes with notably higher expression in C1 than in C2 are also related to tumorigenesis and shown prognostic value (Knox et al., 2009; Sdelci et al., 2019; Wang et al., 2019). Consistent with these findings, the survival rate in the metabolic-enriched group was significantly lower than that in the metabolic-desert group. The evaluation of the SNV profile of the novel two subtypes further demonstrated that *KRAS*, *TP53*, *SMAD4*, and *CDKN2A* are frequently mutated in the C1 subtype. According to Terumi et al., *KRAS*, *TP53*, *SMAD3*, and *CDKN2A* are the key driver genes in PanC (Kamisawa et al., 2016). *KRAS* mutations resulting in the persistent activation of the protein contribute to PanC (Buscail et al., 2020). The KRAS protein could activate several signaling pathways to promote the proliferation, invasion, and migration of cancer cells (Buscail et al., 2020). As a hallmark oncogene, *KRAS* upregulates glycolysis-related enzymes and promotes energy supply in cancer cells, indicating a worse survival (Buscail et al., 2020). Furthermore, *TP53*, *SMAD4*, and *CDKN2A* are frequently mutated genes in malignancies (Alhejaily et al., 2014; Bykov et al., 2018; Ritterhouse et al., 2019). The *KRAS* mutation frequency was significantly higher in the C1 group than in the C2 group, and *KRAS* mutations were associated with increased expression. These findings support the reliability of the novel metabolic subtypes and the association between the metabolism-related gene-enriched group (C1) and a poor prognosis.

We explored the core differences between the subtypes. Using a GSEA, we identified five hallmark gene sets, HALLMARK_E2F_TARGETS, HALLMARK_MYC_TARGETS_ V1, HALLMARK_GLYCOLYSIS, HALLMARK_MTORC1_ SIGNALING, and HALLMARK_NOTCH_SIGNALING, activa ted in the C1 (high risk) group. Repressed by the tumor suppressor gene *RB1* (retinoblastoma susceptibility gene) in RB-E2F complexes, E2F1 induces cell cycle entry and could be a cancer inducer (Fischer and Müller, 2017). Amplification of the *MYC* gene is related to poor outcomes in PanC (Witkiewicz et al., 2015). Additionally, mTORC signaling, glycolysis-related genes, and NOTCH signaling all contribute to the pathogenesis of PanC (Mann et al., 2016; Gao et al., 2017; Yang et al., 2020). And based on network system biology we select one target KRT18 associated with poor prognosis in PanC. KRT18 could induce proliferation, migration, and invasion via MAKP signaling in gastric cancer (Wang et al., 2020). However, the mechanism by which *KRT18* contributes to PanC has not been determined and should be a focus of further research.

Pancreatic cancer (PanC) is a highly lethal malignancy with high complexity and heterogeneity (Yao et al., 2020). The Cancer Genome Atlas Research Network completed integrated genomic, transcriptomic, and proteomic profiling of 150 pancreatic ductal adenocarcinoma specimens. They revealed a complex molecular landscape of pancreatic ductal adenocarcinoma and provided a roadmap for precision medicine (Cancer Genome Atlas Research Network, 2017). Two large researches using PanC samples recently reported gene expression subtypes of PanC, extending the subtypes previously described by Collisson et al. (Collisson et al., 2011; Moffitt et al., 2015; Bailey et al., 2016; Martinez-Useros et al., 2021). Sinkala et al. employed transcriptomic, copy number alteration and mutation profiling datasets from PanC patients together with data on clinical outcomes to show that the three PanC subtypes each display distinctive aberrations in cell signaling and metabolic pathways (Sinkala et al., 2018). Sinkala et al. also identified another two PanC subtypes and biomarker sets that can be used to accurately and sensitively classify novel pancreatic tumors (Sinkala et al., 2020). Inspired by previous researches, we developed novel risk model and metabolic subtypes to deepen our understanding of PanC and to facilitate individualized diagnosis and treatment in clinical settings.

This study had several limitations. First, despite our use of data for large patient cohorts obtained from multiple platforms supporting the reliability and reproducibility of our results, prospective studies are needed before applying the risk model in clinical settings. Additionally, our risk score formula is based on 18 metabolic genes; however, further studies on interactions among these genes and their biological mechanisms are needed.

## Conclusion

In this study, we identified two subtypes, a metabolic gene-enriched subtype (C1) and metabolic gene-desert subtype (C2), in PanC based on the degree of metabolic gene enrichment. The C1 subtype was related to a worse prognosis based a survival analysis and distinct SNVs. We then developed a risk model based on genes associated with the high-risk subgroup (C1) and validated the reliability of the predictive model using an internal validation dataset of TCGA and two external validation datasets. Finally, nine drugs were identified for high-risk patients.

## Data Availability Statement

The datasets presented in this study can be found in online repositories. The names of the repository/repositories and accession number(s) can be found in the article/Supplementary Material.

## Author Contributions

YG and XT were responsible for the design and conception of the research. XF, EZ, LK, PL, and YG contributed to data acquisition or data analysis, and data cleaning. XT and YG participated in the drafting of the manuscript and the rigorous modification of the manuscript to clearly convey the research contents. All authors contributed to the article and approved the submitted version.

## Conflict of Interest

The authors declare that the research was conducted in the absence of any commercial or financial relationships that could be construed as a potential conflict of interest.

## Publisher’s Note

All claims expressed in this article are solely those of the authors and do not necessarily represent those of their affiliated organizations, or those of the publisher, the editors and the reviewers. Any product that may be evaluated in this article, or claim that may be made by its manufacturer, is not guaranteed or endorsed by the publisher.

## Footnotes

1
https://portal.gdc.cancer.gov/

2
https://www.ebi.ac.uk/arrayexpress/

3
https://www.ncbi.nlm.nih.gov/geo/

4
https://www.gsea-msigdb.org/gsea/msigdb

5
https://metascape.org/

6
http://firebrowse.org
