# Supplementary material for: Identification of Novel Metabolism-Associated Subtypes for Pancreatic Cancer to Establish an Eighteen-Gene Risk Prediction Model
Source: Front Cell Dev Biol. 2021 Aug 10;9:691161. doi: 10.3389/fcell.2021.691161 (PMC8383117; doi:10.3389/fcell.2021.691161)
Supplement: Supplementary file 1 [file Data_Sheet_1.PDF]

Supplementary Material

1 **Supplementary Figure 1: (A) Sample overlap for our metabolic subtypes and mRNA subtypes from Moffitt et al., Collisson et al., or Bailey et al. (from outside to inside, respectively); (B) The boxplot showed the difference in the purity of the tumors between the C1 and C2 subtypes. *P* was obtained by t-test, and *P* < 0.05 was defined as statistically significant.**

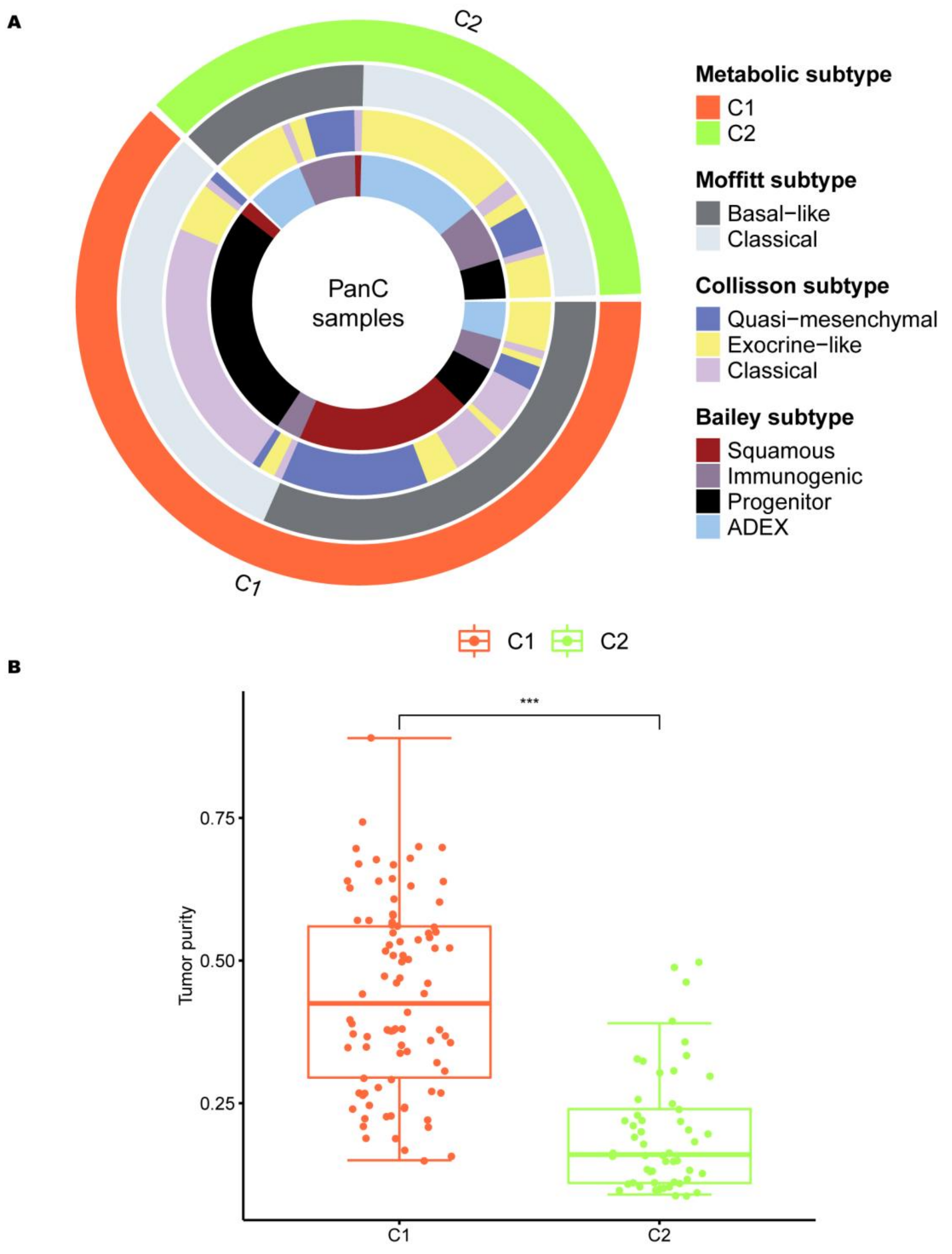

2 Supplementary Figure 2: (A) GO analysis results for C1 subtype. (B) GO analysis results for C2 subtype. (C) KEGG analysis results for C1 subtype. (D) KEGG analysis results for C2 subtype. GO: gene ontology. KEGG: Kyoto Encyclopedia of Genes and Genomes.

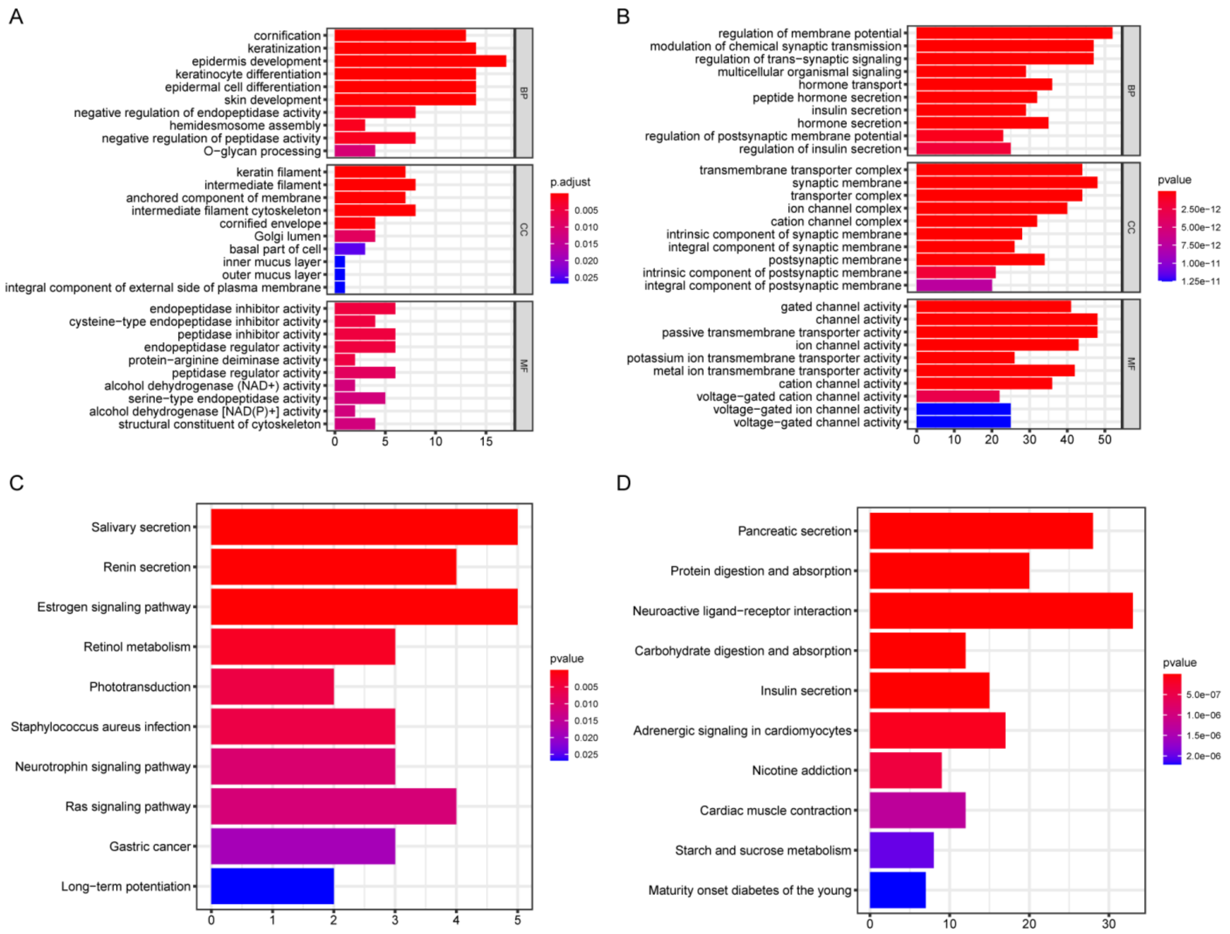

Patient characteristics in the training group and internal validation group are shown in Supplementary Tables 1 and 2.
